# Probiotic Potential, Iron and Zinc Bioaccessibility, and Sensory Quality of *Uapaca kirkiana* Fruit Jam Fermented with *Lactobacillus rhamnosus* Yoba

**DOI:** 10.1155/2020/8831694

**Published:** 2020-12-23

**Authors:** Armistice Chawafambira, Moosa Mahmood Sedibe, Augustine Mpofu, Matthew Achilonu

**Affiliations:** ^1^Department of Agriculture, Faculty of Health and Environmental Sciences, Central University of Technology Free State, Bloemfontein 9300, Private Bag X 20539, South Africa; ^2^Department of Food Science and Technology, Chinhoyi University of Technology, Private Bag 7724, Chinhoyi, Zimbabwe; ^3^Technology Section in Chemical, Mangosuthu University of Technology, Private Bag X 12363, Jacobs, Durban 4026, South Africa

## Abstract

*Uapaca kirkiana* is an underutilised indigenous fruit tree (IFT) found in the miombo ecological zone in sub-Saharan Africa. Furthermore, sub-Saharan Africa is home to many nutritionally insecure people who suffer from micronutrient deficiency. The incorporation of probiotic strains in jams as a possible way of enhancing mineral accessibility, food quality, and health is limited in Africa. This study monitored the probiotic potential, bioaccessible iron and zinc, and organoleptic properties of *U. kirkiana* fruit jam fermented with *L. rhamnosus* yoba. *U. kirkiana* fruits were collected from semiarid rural areas of Zimbabwe. The *L. rhamnosus* yoba strain was obtained from the Yoba for Life Foundation, Netherlands. Mineral and biochemical properties of the probiotic jam were analysed using AOAC standard methods. The *U. kirkiana* fruit tree was ranked first as a food resource by most rural populations in Zimbabwe. The probiotic jam formulation had 55% (wt/vol) *U. kirkiana* fruit pulp, 43% (wt/vol) sugar, 1.25% (wt/vol) pectin, 0.5% (wt/vol) citric acid, and 0.25% (wt/vol) *L. rhamnosus* yoba strain. The probiotic jam had 6.2 ± 0.2 log CFU/mL viable *L. rhamnosus* yoba cells. Iron and zinc content (mg/100 g w.b.) was 4.13 ± 0.22 and 0.68 ± 0.02 with pH 3.45 ± 0.11, respectively. Nutrient content was g/100 g w.b., carbohydrate 66 ± 4.1, fat 0.1 ± 0.01, crude protein 0.2 ± 0.01, ash 0.7 ± 0.02, and crude fiber 0.3 ± 0.01. Bioaccessible iron and zinc were 6.55 ± 0.36% and 16.1 ± 0.50% and increased by 4% and 2% in the probiotic jam, respectively. Mineral bioaccessibility and nutrient content were significantly different (*p* < 0.05) in jam with 0.25% *L. rhamnosus* yoba. Jam acceptance rating was 83%. The probiotic jam can be used as a sustainable food containing probiotic with potential nutritional and health benefits.

## 1. Introduction

*Uapaca kirkiana* belongs to the genus *Uapaca* of the family *Euphorbiaceae* and subfamily *Phyllanthaceae* [1, 2]. The *Uapaca kirkiana* fruit tree is an underutilised indigenous fruit tree (IFT) that is well-adapted to the miombo ecological zone in sub-Saharan Africa [3]. The *U. kirkiana* tree is distributed in semidry and hot areas although it can grow in some relatively wet regions of Zimbabwe [4]. The tree produces fruits which ripen from October to February. The *U. kirkiana* fruit is a good source of sugar, energy, and essential minerals [5–7]. Chawafambira et al. [4] reported that the fruit pulp contains Fe (11.8 mg/100 g FW), Zn (1.3 mg/100 g FW), Ca (17 mg/100 g FW), Mg (39 mg/100 g FW), and K (375 mg/100 g FW). Furthermore, Stadlmayr et al. [8] reported the proximate composition of the fruit as water (72.6 g/100 g), carbohydrates (28.7 g/100 g), proteins (0.5 g/100 g), fat (0.4 g/100 g), calories (523 kcal/kJ), ash (1.1 g/100 g), fiber (2.3 g/100 g), and vitamin C (16.8 mg/100 g).

Bioaccessibility refers to a nutrient fraction that is released from the food matrix and available for absorption in the small intestine [9, 10]. Currently, in vitro assays are mainly used to evaluate mineral bioaccessibility in foods [10] using a simulation model of the digestion process [11]. There is evidence of enhanced bioaccessibility of iron from plant foods caused by household food processing techniques such as heat treatment and fermentation [12]. Fermented foods have a low risk of contamination and provide new desirable taste and texture to food. Fermented products and the contribution of microorganisms provide essential nutrition and health. Fermentation is applied to release the complexed minerals and enhances their bioaccessibility and bioavailability [13]. Food fermentation results in dephytinization leading to increased bioaccessibility and bioavailability of magnesium, iron, calcium, and zinc content [13].

Deficiencies of micronutrients, especially iron and zinc, are nutritional problems that occur the most all over the world [12] and are widely prevalent in most developing countries [10]. Micronutrient deficiencies are often referred to as “hidden hunger” because they are less visible than macronutrient deficiencies [12]. Iron is essential in the synthesis of haemoglobin and myoglobin [14]. Zinc is important in gene regulation and apoptosis [15]. Iron and zinc absorption occurs in the small intestines [16].

Probiotics have been reported to produce short chain fatty acids, which increase the solubility of available calcium [17]. Research by Villa et al. [18] indicated an increase in calcium bioavailability by the action of probiotics such as *Lactobacillus* and *Bifidobacterium* in the hydrolysis of glycoside bonds of estrogenic food in the small intestines. Of late, in vitro bioaccessibility of iron and zinc has been conducted by solubility assays in fruit juices [14].

Many studies have reported the benefits of consuming *L. rhamnosus* GG in the prevention and treatment of upper respiratory tract infections, gastrointestinal infections, and diarrhoea in children [19–21]. The use of *L. rhamnosus* yoba could significantly change sensory attributes, preferences, and acceptability of food by consumers. Mattila-Sandholm et al. [22] noted that sensory aspects of probiotic foods are important in promoting the consumption of functional foods. The effect of *L. rhamnosus* yoba on the mineral bioaccessibility, sensory quality, and chemical properties of fruit jams has not been extensively investigated. Thus, the purpose of this study was to evaluate iron and zinc bioaccessibility and sensory qualities of probiotic *U. kirkiana* fruit jam fermented with *L. rhamnosus* yoba.

## 2. Materials and Methods

### 2.1. Study Area

**Figure 1:**
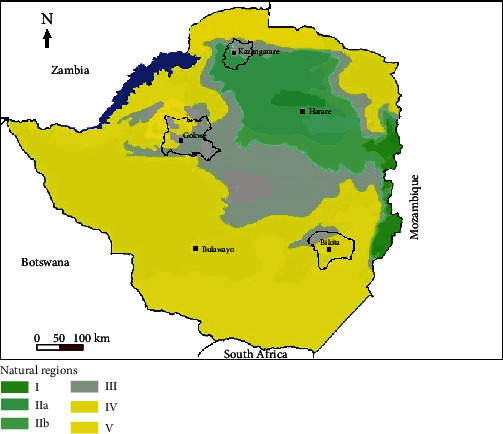
Map showing sampling areas (Bikita, Gokwe, and Kazangarare) of *U. kirkiana* fruits in Zimbabwe.

Ripe *U. kirkiana* fruits were collected from Gokwe (a semidry area located 18.22°S 28.93°E in agro farming region 3), Bikita (a dry area located 20.5°S 31.37°E in agro farming region 4), and Kazangarare (a communal area located 16.30°S 29.56°E in agro farming regions 2b and 3) as shown in Figure 1.

### 2.2. Field Data Collection

Permission to obtain fruit samples was obtained from local leaders (councillors), and consent forms were obtained from participating households.

### 2.3. Focus Group Discussions and Interviews

Focus group discussions and interviews were conducted in the local Shona language for better understanding by all participants. Semistructured questionnaires were used in formal interviews of indigenous people to understand their perceptions on preferences, availability, uses, and consumption of indigenous fruit trees (IFTs). This was important regarding the significance of the *U. kirkiana* fruit tree as a food resource as compared to other indigenous fruit trees. Data collected in focus group discussions and interviews included sociodemographic information such as age and gender. The importance of the *U. kirkiana* fruit as compared to other indigenous fruit trees as a food resource was evaluated by asking participants to rank fruit trees in their area from the most important (ranked 1) to the least important (ranked 5). Data was collected on the consumption pattern and socioeconomic significance of the fruit trees in the areas.

### 2.4. Fruit Collection

**Figure 2:**
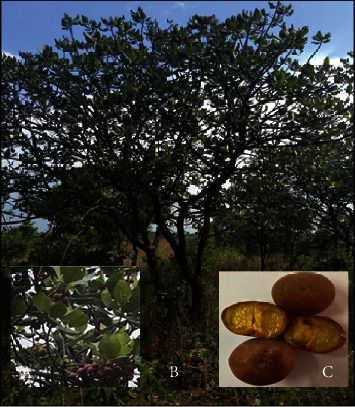
*U. kirkiana* fruits (a), *U. kirkiana* fruit trees (b), and *U. kirkiana* fruit pulp and seed (c).

Fruit trees were chosen randomly using stratified sampling method (Figure 2(b)). Each ward in an area was considered a strata, and a total of 5 wards were considered. Samples of 100 ripe fruits that had fallen from different parts of the tree were randomly collected from the ground (Figure 2(a)). A total of 1000 fruits with a total mass of 8 kg were collected.

### 2.5. Pulp Extraction

Collected ripe fruits were cleaned, and soil particles and stones were removed. The edible pulp was obtained by cutting the fruit, removing seeds, then mashing, and sieving. The crude pulp mixture was sieved through an 800 *μ*M sieve to obtain a composite pulp sample.

### 2.6. Preparation of *U. kirkiana* Fruit Jam

**Figure 3:**
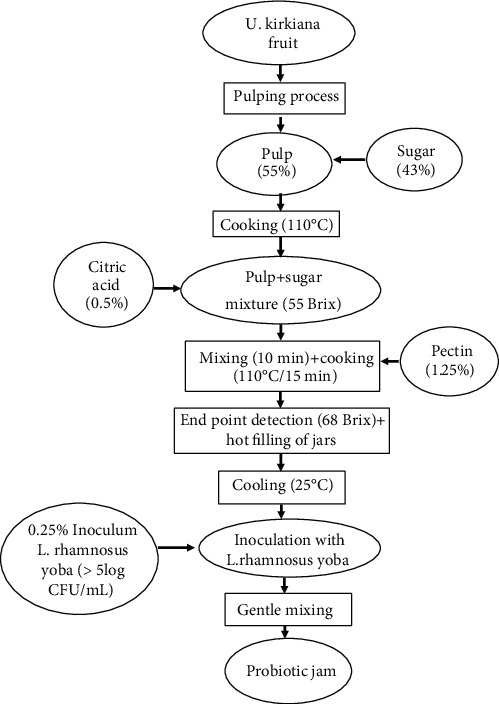
Production process of fruit jam inoculated with *L. rhamnosus* yoba.

The best formulation for making the fruit jam had 55% (wt/vol) composite fruit pulp, 43% (wt/vol) sugar, 1.5% (wt/vol) pectin, and 0.5% (wt/vol) citric acid. In producing the jam, the composite fruit pulp was mixed with sugar in a stainless steel pot and cooked at 110°C until all the sugar had dissolved. Citric acid (0.5%) was added and stirred gently whilst cooking until it reached 55 Brix. Commercial pectin was added, and the mixture was continuously stirred until the jam had reached its end point of 68 Brix as indicated in Figure 3.

### 2.7. Source of *L. rhamnosus* Yoba

*L. rhamnosus* yoba was purchased from the Yoba for Life Foundation, Netherlands, and used in this study. This isolate, *L. rhamnosus* yoba, was obtained from a commercial product containing *L. rhamnosus* GG and was identified and confirmed using 16S rRNA sequencing [21]. This isolate was deposited at the Belgian Coordinated Collections of Microorganisms/Laboratorium voor Microbiologie Gent (BCCM/LMG) culture collection using the name *L. rhamnosus* yoba. This isolate bacterium, *L. rhamnosus* yoba, was purchased and used in this study.

### 2.8. Preparation of Inoculum

Pure strains of *L. rhamnosus* yoba were obtained from the Yoba for Life Foundation, Amsterdam, Netherlands, and stored at −80°C. The *L. rhamnosus* yoba strain was reactivated by subculturing anaerobically in de Man, Rogosa, and Sharpe (MRS) agar broth at 37°C for 18 hours. The fruit pulp was mixed with sugar, boiled, and subsequently cooled to room temperature (25°C). Five grams of *L. rhamnosus* yoba pure strain was then precultured in the medium and incubated at 37°C for 36 hours. Growth of the bacterium was monitored until the number of live cells was more than 6 log CFU/mL.

### 2.9. Inoculation of Probiotic Culture into the Jam

Sterilized bottles (400 ml) containing *U. kirkiana* fruit jam (100 g) were opened under aseptic conditions, and the jam was inoculated with 2.5 mL of fresh probiotic culture. *L. rhamnosus* yoba cell suspensions of the culture were gently mixed with the jam. In the control experiment, distilled water (2.5 mL) was boiled, cooled to 30°C, and inoculated into the fruit jam.

### 2.10. Determination of Growth Rate of *L. rhamnosus* Yoba

The growth rate of *L. rhamnosus* yoba in the jam was evaluated over a period of 24 hours. Sampling was done every 2 h over the 24 h period. One milliliter (1 mL) of a sample was aseptically taken from the jam and suspended in sterile 9 ml of peptone physiological salt solution (pH 7.0, 8.5 g/L NaCl, and 1 g/L neutralized bacteriological peptone from Oxoid). Diluents of 100 *μ*L were plated in triplicate onto de Man, Rogosa, and Sharpe (MRS) agar (1.2% agar, bacteriological peptone from Oxoid, added to de Man, Rogosa, and Sharpe broth, Merck). MRS agar plates were incubated at 37°C under anaerobic conditions in GasPak anaerobic jars (Becton Dickinson Microbiology Systems, Baltimore, Maryland, USA). All colonies on the MRS agar were counted, and the results were expressed as colony forming units per milliliter (CFU/mL) of *L. rhamnosus* yoba, taking into account the dilution factors.

### 2.11. Iron and Zinc *In Vitro* Bioaccessibility Assay

Iron and zinc bioaccessibility were determined using the INFOGEST *in vitro* digestion protocol [23]. The initial iron and zinc content in the probiotic jam was measured before and after the oral, gastric, and intestinal phases of simulated gastrointestinal digestion.

#### 2.11.1. Oral Phase

A jam sample of 5 g was mixed with 4 mL of simulated salivary fluid (SSF). To this sample, 0.95 mL of Milli-Q water was added, followed by the addition of 25 *μ*L of CaCl_2_ solution and 25 *μ*L of *α*-amylase (75 units/mL). The resultant mixture was incubated for 2 min at 37°C in a shaking water bath.

#### 2.11.2. Gastric Phase

In the simulated digestion phase, 7.5 mL of simulated gastric fluid (SGF), 1.6 mL pepsin solution (2000 units/mL), and 5 *μ*L of CaCl_2_ solution were added to the mixture from the oral phase. The pH of the mixture was adjusted to 3 by adding approximately 0.8 mL of 6 M hydrochloric acid. The resultant mixture was incubated for >2 min at 37°C in a shaking water bath.

#### 2.11.3. Intestinal Phase

In the intestinal phase of simulated digestion, solutions were added in the following sequence to the mixture from the gastric phase; 11 mL of simulated intestinal fluid (SIF), 5 mL of pancreatin solution (100 units/mL), 2.5 mL of bile solution (10 mM), and 40 *μ*L of CaCl_2_. The pH of the mixture was adjusted to 7 by adding 1 M NaOH dropwise (0.5 mL), and the mixture was incubated for 2 hours at 37°C in a shaking water bath at 300 rpm. A sample of 1 mL was collected after simulated intestinal digestion and pipetted into capped microcentrifuge tubes. The sample was then analysed for the zinc and iron content.

### 2.12. Zinc and Iron Analyses

Iron and zinc content was determined using an Inductively Coupled Plasma-Optical Emission Spectrometer (ICP-OES) (Agilent 5100, Agilent Technologies, Santa Clara, California, USA), which allows for simultaneous detection of minerals [24]. Samples of the probiotic jam and/or intestinal phase sample were digested using concentrated solutions of nitric acid (HNO_3_) and sulphuric acid (H_2_SO_4_), followed by addition of ultrapure hydrogen peroxide (H_2_O_2_) to complete the digestion. Residual samples were filtered off where necessary. The digested samples were then fed into the automated ICP-OES by vacuum-operated pipes, and results were recorded. Results were converted from ppm per 100 g to mg per 100 g fresh mass (FM) by dividing by 10.

### 2.13. Iron and Zinc Bioaccessibility Calculation

Iron and zinc content results from the ICP-OES analysis were used to calculate the bioaccessibility of the minerals according to an equation adopted from Hemalatha et al. [25].

(1) $$\begin{array}{c} \text{Bioaccessibility }\left(\%\right)=100\times \frac{Y}{Z}, \end{array}$$

where *Y* is the element content of the bioaccessible fraction (mg mineral/100 g) and *Z* is the total mineral (zinc or iron) content (mg/100 g).

### 2.14. Biochemical Analysis

Crude protein, fat, ash, and crude fiber content of the probiotic jam were determined according to Association of Official Analytical Chemistry [24] methods. Total carbohydrate content of probiotic jam was estimated by the difference method.

### 2.15. pH Measurement

The pH was determined according to the AOAC standard method using a digital pH meter (BT-675, BOECO, Hamburg, Germany). The glass electrode of the pH meter was calibrated using standard buffer solutions (pH 4 and pH 7) prior to pH measurements [26].

### 2.16. Sensory Evaluation Process

In the sensory evaluation process, 140 taste panels were selected using systematic random sampling. Demographic information about the taste panelists was collected. Consent forms were signed by each panelist. A sensory questionnaire using a 9-point hedonic scale and preference test was designed using Shona, a local language used by the panelists. The 9-point hedonic scale used the following key: 1: dislike extremely, 2: dislike very much, 3: dislike moderately, 4: Dislike slightly, 5: neither like nor dislike, 6: like slightly, 7: like moderately, 8: like strongly, and 9: like extremely; instructions for the panelists was translated in the local language for easy understanding of the sensory process. The panelists evaluated mouth feel, taste, aroma, texture, spreadability, and overall acceptance and rated their responses on the 9-point hedonic scale. A commercially made mixed fruit jam was used a benchmark product, and a paired preference test was used to compare the taste. Panelists were not allowed to discuss their results during the sensory evaluation process. Panelists were presented with a jam sample weighing 25 g each. Samples were served in small paper plates covered with aluminum foil.

#### 2.16.1. Triangle Test

The ability of trained and untrained panelists to discriminate between probiotic jam samples and ordinary jam samples was calculated using a triangle test. The panelists were drawn from three areas, Gokwe (40), Bikita (40), and Kazangarare (40), and trained panelists were from the Department of Food Science and Technology, Chinhoyi University of Technology (20). Temporary sensory testing booths made of cardboard box were used by untrained panelists in Gokwe, Bikita, and Kazangarare. The jam samples were coded as A1B, 1AB, 1BA, B1A, BA1, and AB1. The coded samples were randomly given to each panelist together with a glass of water to rinse their mouth. The panelists were evaluated on their ability to discriminate differences in appearance and taste of the jam samples. The panelists who were able to discriminate the taste of the jam samples for both the trained and untrained panelists were used in the preference test.

#### 2.16.2. Preference Test

Trained panelists were used to indicate their preference on sweetness, color, aroma, texture, and overall acceptance of the jams using a 9-point hedonic scale.

### 2.17. Statistical Analysis

The results of zinc and iron analyses, probiotic viability, pH, and sensory properties were expressed as the mean ± standard deviation (SD), and all experiments were conducted in triplicates. LSD test was conducted to determine any significant differences at *p* < 0.05. Customer acceptability and sensorial results were analysed using one-way Analysis of Variance (ANOVA). Probabilities for triangle taste tests were computed to analyse the triangular taste test data. All the analyses were done using SPSS package version 18.0 (Coakes and Ong, John Wiley & Sons, Queensland, Australia).

## 3. Results and Discussion

The sample areas, Gokwe and Bikita, are farming communities in the semiarid and hot agroecological region of Zimbabwe. The *U. kirkiana* tree is well adapted to hot, dry areas [4].  Table 1 shows the importance of the *U. kirkiana* fruit tree as a food resource for the rural population in relation to other indigenous fruit trees (IFTs). The *U. kirkiana* was ranked first in Bikita and Gokwe, mainly because of its significant use and perceived nutritional benefits. Fruit pulp is eaten raw, and the seeds and skin are removed. *U. kirkiana* fruits are sold on the roadside and informal and formal markets to generate income. This observation was supported by Chawafambira et al. [4].

### 3.1. Chemical Composition

The nutrient content of the probiotic fruit jam in g/100 g fresh weight basis was carbohydrate 66 ± 4.1, fat 0.1 ± 0.01, crude protein 0.2 ± 0.01, ash 0.7 ± 0.02, and crude fiber 0.3 ± 0.01 (Table 2). The probiotic jam is a good source of dietary iron and zinc and can possibly be used to supply these essential minerals in the body. Mineral deficiency is prevalent in most rural and urban areas in sub-Saharan Africa. Chawafambira et al. [4] reported that the *U. kirkiana* fruit is a good source of iron (11.8 mg/100 g), calcium (17 mg/100 g), potassium (375 mg/100 g), magnesium (39 mg/100 g), and phosphorus (15 mg/100 g). The high carbohydrate content makes the jam a good energy providing food.

### 3.2. Bioaccessible Iron and Zinc

Jam that was inoculated with *L. rhamnosus* yoba had an iron bioaccessibility of 6.55 ± 0.36% (Table 3). Iron bioaccessibility was significantly different from that of the control jam (*p* < 0.05). Zinc bioaccessibility of the *L. rhamnosus* yoba jam was significantly different from that of the control jam (*p* < 0.05). The jam inoculated with *L. rhamnosus* yoba had a zinc bioaccessibility of 16.1 ± 0.50% (Table 4). Iron plays an important role in the human body, particularly in the formation of red blood cells. Iron bioaccessibility of probiotic (*L. rhamnosus* yoba) jam increased by 4% when the jam was inoculated with *L. rhamnosus* yoba. Zinc bioaccessibility in probiotic and control jams was 16.1 ± 0.5% and 14 ± 0.33%, respectively. This translated to an increase of 2% in zinc bioaccessibility when *L. rhamnosus* yoba was inoculated into the jam. This could be attributed to the action of *L. rhamnosus* yoba as it produced degradation enzymes that acted on the food matrix to release the bound zinc.

Furthermore, the effect of processing during jam-making may cause the breakdown of complex polysaccharides from the food matrix under the action of pectinase to release the bound minerals. Khouzam et al. [27] reported a bioaccessibility of 6.7–12.7% for essential minerals in different fruits and vegetables. The bioaccessibility of iron might have been affected by the presence of inhibiting compounds such as phytates and carbonate salts during fruit maturation, which may chelate and form insoluble complexes with iron resulting in reduced iron bioaccessibility [27]. In its structure, phytic acid contains an inositol ring with 6 phosphate ester groups, and it chelates iron and zinc ions, forming insoluble complexes in the upper gastrointestinal tract [28]. These complexes cannot be digested or absorbed due to the absence of intestinal phytase enzymes in humans [29]. The analysis for phytic acid was not reported in this study, and this could not quantify the amount of phytic acid present in the jam; hence, an assumption was used explain its effect on the iron and zinc bioaccessibility. Furthermore, there is need to carry out an assay to determine the amount of phytic acid in the fruit pulp and jam.

*U. kirkiana* fruits contain organic acids such as malic and oxalic acids, and these might have complexed the iron and zinc during fruit maturation. Phenolic compounds in fruits reduce mineral bioaccessibility. The *U. kirkiana* fruit pulp had a total phenolic content of 67–82.5 *μ*g GAE/g [30]. The presence of phenolic compounds in the fruit could explain the low bioaccessibility of iron.

Zinc is an essential micronutrient in the human body and is involved in many metabolic processes catalyzed by different enzymes. Its deficiency may lead to retarded growth and dermatitis [31]. The Zimbabwe Demographic and Health Survey (ZDHS) [32] report states the RDA for zinc and iron as 3–11 mg/100 g and 13–19 mg/100 g, for age groups 1-9 years and 9-13 years, respectively. Therefore, calculations on % contributions on RDA for essential minerals have indicated that the jam has a potential to deliver more than 20% and 30% of the RDA for iron and zinc in age groups 1-9 years and 9-13 years, respectively.

During the pulping process, the action of pectinase might have resulted in the release of zinc from the pectin matrix in the fruit pulp. The *U. kirkiana* fruit contains relatively high levels of calcium [4], and calcium has been found to inhibit the bioaccessibility of other minerals such as zinc [33]. Phytates that build up in the fruit pulp during the maturation process could have affected zinc bioaccessibility [34].

**Figure 4:**
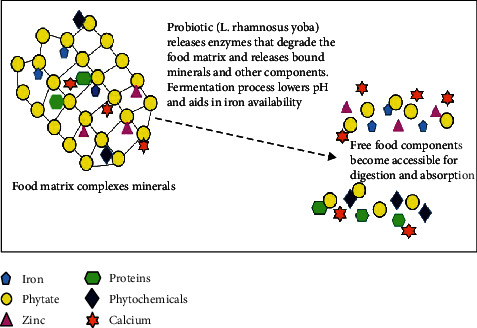
Possible *L. rhamnosus* yoba mechanism in increased mineral bioaccessibility (adopted from Sripriya et al. [35]).

The possible mechanism by which *L. rhamnosus* yoba leads to increased mineral bioaccessibility and/or bioavailability was designed and represented by the author in Figure 4.

Conversely, the loss of dry matter during fermentation increases mineral content as lactic acid bacteria degrades sugar and protein [36]. *L. rhamnosus* yoba is capable of fermenting glucose into organic acids in the jam. The observed increase in iron content could be explained by the process of fermentation which degrades phytates that complex with minerals thereby releasing the iron and other minerals such as zinc, calcium, and phosphorous in the jam [35]. Lopez et al. [37] reported the importance of fermentation in reducing phytic acid that binds minerals and making them free and more bioaccessible and available for absorption in the human body. The probiotic, *L. rhamnosus* yoba, could have produced *α*-amylase enzymes that loosen the food matrix by degrading starch. Liang et al. [38] reported that fermenting microorganisms are able to degrade fiber and loosen the bound minerals in the food matrix. Chawafambira et al. [4] reported a fiber content of 2 g/100 g in the *U. kirkiana* fruit pulp. Furthermore, *L. rhamnosus* yoba might have degraded the fiber and released bound minerals in the jam. As the *L. rhamnosus* yoba was fermenting sugars into organic acids, the pH of the jam decreased to 3.45 and enhanced mineral accessibility. At such a low pH, absorption of iron increases because of the conversion of ferrous iron to ferric iron, which is more bioaccessible and available for absorption [39].

### 3.3. Enumeration of *L. rhamnosus* Yoba and pH in Probiotic Jam

Viable counts of *L. rhamnosus* yoba in the jam were determined at *t* = 0 (time of inoculation) and end of incubation period after 24 hours (Table 5). The produced *U. kirkiana* fruit jam was inoculated with *L. rhamnosus* yoba at a level of 5.6 ± 0.1 log CFU/mL. Mpofu et al. [40] reported an *L. rhamnosus* yoba level of 5.8 ± 0.3 log CFU/mL in inoculum that was used in the production of probiotic *mutandabota*. The viable plate count of *L. rhamnosus* yoba increased to 6.2 ± 0.2 log CFU/mL after incubation for 24 h at 37°C. This suggests that the jam matrix was an ideal environment to support the growth of *L. rhamnosus* yoba. More so, Wood and Holzapfel [41] reported *L. rhamnosus* yoba, as a mesophile that grows at a wide temperature range of 15–40°C. Stadlmayr et al. [8] reported proximate composition of the *U. kirkiana* fruit pulp on a dry wet basis as water (72.6 g/100 g), crude protein (0.5 g/100 g), fat (0.4 g/100 g), ash (1.1 g/100 g), fiber (2.3 g/100 g), vitamin C (16.8 mg/100 g), and carbohydrate (28.7 g/100 g).

The sugars in the fruit pulp plus added table sugar acted as sources of carbon that were apparently enough to support the growth of *L. rhamnosus* yoba. Ahmed and Mital [42] explained the simulated growth of a related lactic acid bacterium, *L. acidophilus*, caused by the presence of simple sugars, mainly glucose and fructose, and the minerals manganese and magnesium which are growth promoters. Minerals reported in the *U. kirkiana* fruit pulp (mg/100 g FW) are iron 11.8, magnesium 39, calcium 17, potassium 375, sodium 10, zinc 1.3, phosphorus 15, and copper 0.1 [4, 8, 43].

The relationship between the jam pH and storage time is shown in Table 5. At inoculation (*t* = 0), the pH of the fruit jam was 3.5 ± 0.12. This pH allowed the growth of *L. rhamnosus* yoba in the jam as explained by the increase in cell numbers. The pH of the inoculated jam decreased with time and reached 3.45 at the end of the incubation period. This decrease in pH allowed a survival of the *L. rhamnosus* yoba, but its rate of growth was limited. This supported the growth of *L. rhamnosus* yoba from 5.4 ± 0.1 to 6.2 ± 0.2 log CFU/m in the jam. The low pH could be attributed to citric acid and the presence of naturally occurring organic acids in the fruit pulp such as citric, malic, tartaric, succinic, and ascorbic acids [44, 45]. Citric acid is mainly added at a low concentration to balance and improve the pH in jam making [46]. After 24 h, the pH had decrease to 3.45 ± 0.11. More so, as *L. rhamnosus* yoba was growing and increasing in cell numbers, it was able to ferment sugars into lactic acid and caused a decrease in pH. The low pH (3.45) observed in the jam has the potential to inhibit growth of most pathogenic bacteria, thus ensuring a microbiologically safe probiotic jam, although there is need to conduct an assay on the occurrence of pathogenic bacteria in the jam. This is supported by the International Commission on Microbiological Specifications for Foods [47] which reported that at such a low pH of 3.45, most pathogens do not survive or grow. Liew et al. [48] indicated that the optimum pH for growth of *L. rhamnosus* is pH 6.4 to 6.9. The lowest pH for growth of the lactic acid bacterium is within the range of pH 4.4-3.4. Our results for pH in all jam samples were able to support growth of *L. rhamnosus* yoba although they were out of the optimum range for the bacteria. This could be a result of addition of citric acid used to balance the pH of the jam and promote better gel formation of the jam.

### 3.4. Demographic Information of Sensory Panelists

**Figure 5:**
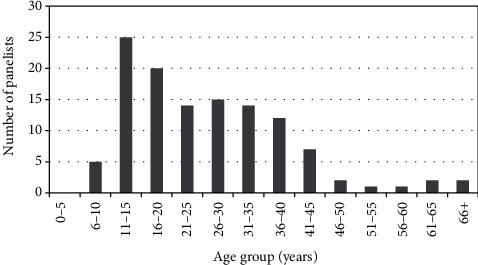
Age distribution of panelists.

The probiotic jam can be consumed by populations of all age groups in rural communities as indicated in Figure 5. Age groups 11–15 yrs., 16–20 yrs., and 26–30 yrs. had 25, 20, and 15 panelists, respectively. The age group 66+ yrs. had 1 participant. The age group 0–5 yrs. had no participants because they could not comprehend the sensory evaluation process. The sensory evaluation was performed by panelists with minimum years of 10. The population distribution in the sample areas indicated that age groups 11–15 and 16–20 yrs. were the most common as compared to older ages [49, 50]. The gender distribution of the panelists was 71 women and 49 men. Rural areas in Zimbabwe have more women than men due to rural exodus, drifting most men to urban areas in search of employment [51].

### 3.5. Triangle and Preference Test

**Figure 6:**
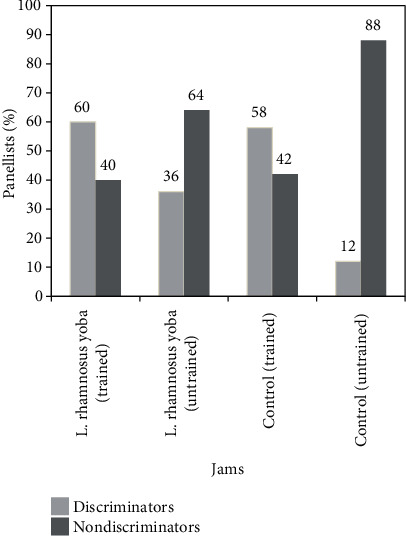
Triangle test (discrimination between *L. rhamnosus* yoba and control jams).

**Figure 7:**
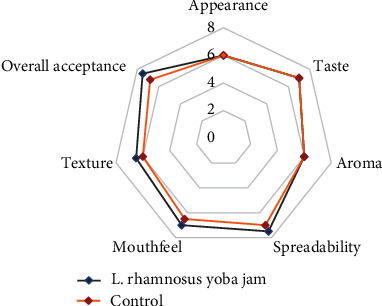
Preference test on jam inoculated with *L. rhamnosus* and control sample.

There was a significant difference (*p* < 0.05) between the trained and untrained panelists with respect to their ability to discriminate the taste of the probiotic and the control jam. Using a triangle test, 60% and 36% of the trained and untrained panelist were able to discriminate the probiotic jam, respectively, using taste and appearance. The triangle test showed that 58% and 12% of the trained and untrained panelist were able to discriminate the control jam, respectively. Forty percent (40%) and 74% of the trained and untrained panelists were unable to discriminate the probiotic jam, respectively (Figure 6). Forty-two (42%) percent and 88% of the trained and untrained panelists were unable to discriminate the control jam, respectively. The panelists that showed the ability to discriminate conducted the paired preference test. The probiotic jam had a mean preference score of 7.5 out of 9, 6.5 out of 9, and 7 out of 9 for spreadability, texture, and mouth feel, respectively (Figure 7). The mean sensory scores were significantly different using LSD test at the 5% level. The probiotic jam had an overall acceptance score of 7.5 out of 9. It was noted that aroma, taste, and appearance were not significantly different (*p* < 0.05) in all jam samples. This is supported by the panelist scores for taste, appearance, and aroma (Figure 7). The main sensory descriptors used on the probiotic jam were “a sweet taste” and “excellent spreadability” by over 80% of the panelists.

There was a significant difference in triangle test results (*p* < 0.05). The trained panelists exhibited a significantly higher success rate of 60% compared to 40% for the untrained panelists with respect to correct identification of the probiotic jam. This could be attributed to the fact that the training and experience of the panelists influenced the proper discrimination of the samples during evaluation.

## 4. Conclusion

*Lactobacillus rhamnosus* yoba was successfully propagated in the *U. kirkiana* fruit jam to produce a probiotic food. The jam had 5.4 ± 0.1 log CFU/mL viable cells on the onset of incubation and 6.2 ± 0.2 log CFU/mL viable *L. rhamnosus* yoba cells on consumption. The fruit jam met the criterion of being a probiotic food because it contained >6 log CFU/mL viable *L. rhamnosus* yoba cells upon consumption with a possibility of promoting health in the human body. Furthermore, the *U. kirkiana* jam was able to support the growth of *L. rhamnosus* yoba to a level of 6.2 log CFU/mL viable cells on the point of consumption thereby meeting the criteria of being a probiotic food. The probiotic jam (with *L. rhamnosus* yoba) had an iron bioaccessibility of 6.55 ± 0.36% and a zinc bioaccessibility of 16.1 ± 0.50%. The use of *L. rhamnosus* yoba increased iron and zinc bioaccessibility by 4% and 2%, respectively. The probiotic jam is potentially an excellent source of iron and zinc. The probiotic jam had a low pH which ensured its microbiological safety although there is need to evaluate its safety. The findings of sensory evaluation indicated that the probiotic jam had good organoleptic properties. A paired difference test showed a significant difference (*p* < 0.05) in jam preferences. More so, the taste of probiotic jam was significantly different from the benchmark sample (mixed fruit jam). The probiotic jam had a higher mean acceptance score of 7.5 using a hedonic rating scale, and this translated to an 83% acceptance rating by the panelists. The attributes that contributed to a high acceptability of probiotic jam were mouth feel (7.5 out of 9), spreadability (7.5 out of 9), and texture (6.5 out of 9). This study will provide more insights on the need to use *L. rhamnosus* yoba in fermenting food materials as a way to enhance mineral bioaccessibility. Furthermore, the propagation of *L. rhamnosus* yoba in the fruit creates the need to use other indigenous fruit trees and promote access to beneficial probiotic by most poor populations in sub-Saharan Africa. The study recommends further research on the effect of *L. rhamnosus* yoba action on inactivation of pathogenic microbes and product shelf life.

## Figures and Tables

**Table 1:** Ranking of indigenous fruit trees (IFTs) as food resources by locals in Gokwe, Bikita, and Kazangarare, Zimbabwe.

Local name	Botanical name	Family	Use	Rank	Consumption (%)
**Gokwe communal area**					
Wild loquat (Eng), mushuku/muzhanje (Sh), umhobohobo (Nd)	*Uapaca kirkiana*	Euphorbiaceae	Fruit eaten raw without seeds	1	70
Monkey orange (Eng), mutamba (Sh), umkhemeswane (Nd)	*Strychnos cocculoides*	Strychnaceae	Fruit eaten raw	2	55
Bird plum (Eng), munyii (Sh), umnyiyi (Nd)	*Berchemia discolor*	Rhamnaceae	Fruit pulp eaten raw	3	45
Chocolate berry (Eng), mutsubvu (Sh), umtshwankela (Nd)	*Vitex payos*	Lamiaceae	Fruit eaten raw	4	40
Snot apple (Eng), mutohwe (Sh)	*Azanza garckeana*	Malvaceae	Fruit is chewed raw without seeds	5	35
**Kazangarare communal area**					
Chocolate berry (Eng), mutsubvu (Sh), umtshwankela (Nd)	*Vitex payos*	Lamiaceae	Fruit pulp eaten raw	1	74
Wild loquat (Eng), mushuku/muzhanje (Sh), umhobohobo (Nd)	*Uapaca kirkiana*	Euphorbiaceae	Fruit eaten raw	2	70
Bird plum (Eng), munyii (Sh), umnyiyi (Nd)	*Berchemia discolor*	Rhamnaceae	Fruit pulp eaten raw	2	62
Snot apple (Eng), mutohwe(Sh)	*Azanza garckeana*	Malvaceae	Fruit is chewed raw without seeds	3	47
Monkey orange (Eng), mutamba (Sh), umkhemeswane (Nd)	*Strychnos cocculoides*	Strychnaceae	Fruit eaten raw	4	40
Bakota plum (Eng); munhunguru (Sh), umqokolo (Nd)	*Flacourtia indica*	Salicaceae	Fruit is eaten raw	5	35
**Bikita communal area**					
Wild loquat (Eng), mushuku/muzhanje (Sh), umhobohobo (Nd)	*Uapaca kirkiana*	Euphorbiaceae	Fruit eaten raw	1	76
Chocolate berry (Eng), mutsubvu (Sh), umtshwankela (Nd)	*Vitex payos*	Lamiaceae	Fruit pulp raw	1	60
Monkey orange (Eng), mutamba (Sh), umkhemeswane (Nd)	*Strychnos cocculoides*	Strychnaceae	Fruit eaten raw	2	53
Bird plum (Eng), munyii (Sh), umnyiyi (Nd)	*Berchemia discolor*	Rhamnaceae	Fruit pulp eaten raw	3	51
Bakota plum (Eng), munhunguru (Sh), umqokolo (Nd)	*Flacourtia indica*	Salicaceae	Fruit eaten raw	4	48
Waterberry (Eng), mukute (Sh)	*Syzygium cordatum*	Myrtaceae	Fruit eaten raw	4	45
Mobola plum (Eng), muhacha/muchakata (Sh)	*Parinari curatellifolia*	Chrysobalanaceae	Fruit pulp eaten raw	5	36

Key: Eng: English; Sh: Shona; Nd: Ndebele.

**Table 2:** Mineral and nutrient content of probiotic and control fruit jam per 100 g wet basis.

Nutrient (g)	Probiotic jam (inoculated with *L. rhamnosus* yoba)	Control
Carbohydrates	66.0 ± 4.1^a^	65.2 ± 2.3^b^
Crude protein	0.2 ± 0.01^a^	0.1 ± 0.01^b^
Fat	0.1 ± 0.01^a^	0.1 ± 0.01^a^
Crude fiber	0.3 ± 0.01^a^	0.1 ± 0.01^a^
Ash	0.7 ± 0.02^a^	0.4 ± 0.01^b^
*Minerals (mg)*		
Iron	4.13 ± 0.22^a^	3.10 ± 0.10^b^
Zinc	0.68 ± 0.02^a^	0.42 ± 0.01^b^

Mean ± standard deviations are reported. Means with identical superscripts in a row are not significantly different at *p* < 0.05.

**Table 3:** *In vitro* digestion on iron content of the jam inoculated with *L. rhamnosus* yoba.

Iron content (mg/100 g FM)				
Sample	Undigested (total content)	After digestion	Bioaccessible portion	Bioaccessibility (%)
*L. rhamnosus* yoba jam	4.13 ± 0.22^a^	3.86 ± 0.14^b^	0.27 ± 0.08^a^	6.55 ± 0.36^a^
Control	4.03 ± 0.41^a^	3.92 ± 0.03^b^	0.11 ± 0.38^b^	2.7 ± 0.92^b^

Mean ± standard deviations are reported. Means with identical superscripts in a column are not significantly different at *p* < 0.05.

**Table 4:** *In vitro* digestion on zinc content of the jam inoculated with *L. rhamnosus* yoba.

Zinc content (mg/100 g FM)				
Sample	Undigested (total content)	After digestion	Bioaccessible portion	Bioaccessibility (%)
*L. rhamnosus* yoba jam	0.68 ± 0.02^a^	0.57 ± 0.01^a^	0.11 ± 0.01^a^	16.1 ± 0.50^a^
Control	0.64 ± 0.03^b^	0.55 ± 0.02^a^	0.09 ± 0.01^b^	14.0 ± 0.33^b^

Mean ± standard deviations are reported. Means with identical superscripts in a column are not significantly different at *p* < 0.05.

**Table 5:** Viability of *L. rhamnosus* yoba and pH in jam.

Time (hours)	Viable cells (log CFU/mL)	pH	
		Probiotic jam	Control
Inoculation: *t* = 0	5.4 ± 0.1	3.50 ± 0.12^a^	3.50 ± 0.12^a^
4	5.5 ± 0.3	3.50 ± 0.10^a^	3.50 ± 0.10^a^
8	5.7 ± 0.2	3.48 ± 0.10^a^	3.50 ± 0.10^a^
12	5.8 ± 0.1	3.47 ± 0.11^a^	3.50 ± 0.01^a^
16	5.8 ± 0.1	3.46 ± 0.10^a^	3.49 ± 0.01^b^
20	6.0 ± 0.1	3.45 ± 0.12^a^	3.48 ± 0.01^b^
End of incubation: *t* = 24	6.2 ± 0.2	3.45 ± 0.11^a^	3.47 ± 0.01^b^

Mean ± standard deviations are reported. Means with identical superscripts in a row are not significantly different at *p* < 0.05.

## Data Availability

The data of this research is available and will be provided upon request.
